# Effects of Functional Impairments and Frailty on the Association of Cognitive Impairment with Total Healthcare Costs: A Prospective Multi-cohort Study

**DOI:** 10.1007/s11606-025-10073-z

**Published:** 2025-12-09

**Authors:** Howard A. Fink, Kerry M. Sheets, Lisa Langsetmo, John T. Schousboe, Allyson M. Kats, Kristine E. Ensrud

**Affiliations:** 1 Geriatric Research Education and Clinical Center, Veterans Affairs Health Care System, Minneapolis, MN, USA; 2 Center for Care Delivery & Outcomes Research, Veterans Affairs Health Care System, Minneapolis, MN, USA; 3 Department of Medicine, University of Minnesota, Minneapolis, MN, USA; 4 Division of Epidemiology & Community Health, School of Public Health, University of Minnesota, Minneapolis, MN, USA; 5 Department of Medicine, Hennepin Health, Minneapolis, MN, USA; 6 HealthPartners Institute, Bloomington, MN, USA; 7 Division of Health Policy & Management, School of Public Health, University of Minnesota, Minneapolis, MN, USA

**Keywords:** Cognitive impairment, Dementia, Health care costs, Frailty, Functional status

## Abstract

**BACKGROUND:**

Cognitive impairment is associated with higher total healthcare costs (THC) after accounting for demographic factors and comorbidity burden.

**OBJECTIVE:**

Determine the proportion of incremental THC associated with cognitive impairment that is accounted for by functional impairments and frailty.

**DESIGN:**

Four prospective cohort studies of community-dwelling older adults are linked with each other and Medicare claims.

**PARTICIPANTS:**

Eight thousand one hundred sixty-five community-dwelling, fee-for-service Medicare beneficiaries (mean age 79 years, 53% women, 79% non-Hispanic White).

**MAIN MEASURES:**

Cognitive impairment was defined by either abnormal cognitive tests or self-or-proxy report of a clinician diagnosis of dementia. Comorbidity was the count of claims-based chronic conditions. Functional impairments were self-reported difficulty performing four activities of daily living. Frailty was defined by the Cardiovascular Health Study phenotype or a claims-based deficit accumulation index (CFI). Annualized THC were ascertained for 36 months following an index exam. Incremental THC of cognitive impairment were mean THC in women (men) with cognitive impairment minus the mean THC in women (men) without cognitive impairment.

**KEY RESULTS:**

Incremental THC of cognitive impairment after adjustment for age, race, geographic region, and comorbidities was $6883 (95% CI, 3461–10,305) in women and $7276 (3298–11,254) in men. A substantial proportion of incremental THC of cognitive impairment were attributable to functional impairment, phenotypic frailty, or CFI individually (range 31.1–40.2% in women and 27.6–40.1% in men), while the combination of these three domains accounted for the majority of incremental THC (63.4% in women and 58.2% in men).

**CONCLUSIONS:**

For both women and men, after accounting for demographics and comorbidity, approximately 60% of incremental THC associated with cognitive impairment was attributable to functional impairments and frailty. Investigation is warranted to determine if addressing these geriatric syndromes will mitigate their impact on THC among community-dwelling older adults with cognitive impairment.

**Prior Presentations:**

Results included in this manuscript were previously presented at the Gerontological Society of America Scientific Meeting in Seattle, WA, on November 16, 2024.

## Graphical Abstract

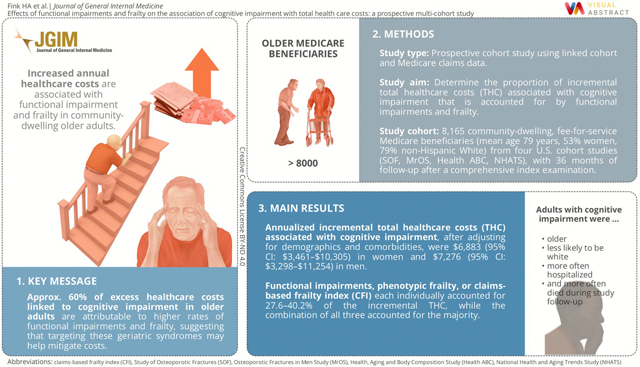

## INTRODUCTION

Dementia is a clinical syndrome in which acquired cognitive deficits interfere with independence in activities of daily living.^1^ Frailty is a clinically recognizable state of diminished physiological reserve and increased vulnerability to adverse health outcomes.^2^ Functional impairment is a broad concept that may refer to impairment in body functions, activities, and participation.^3^

In the USA, dementia prevalence is about 10% in adults aged 65 years or older.^4–6^ It is established that dementia is costly to patients, families, and society,^7–9^ and many factors associated with these costs have been investigated. In two systematic reviews and more recent US studies, among adults with dementia near the end of life, minoritized race or ethnicity and greater comorbidity are consistently associated with higher healthcare costs and utilization, while results are mixed for female sex.^10–13^ Among US studies in individuals with dementia not focused on end of life care, results suggest costs and utilization are higher with increased functional impairments, but results are mixed for increased comorbidities and greater cognitive impairment or dementia severity.^14–18^ We found no studies that specifically examined the effect of frailty on the association of dementia with healthcare costs or utilization.^11^ In addition, most studies on factors associated with dementia costs were limited by retrospective or cross-sectional design or by not comparing results to a non-dementia control group.

In recently published prospective analyses utilizing pooled data from four multi-site US cohort studies linked with Medicare claims, we found that cognitive impairment (self-or-proxy-reported clinician diagnosis of dementia or impairment on brief cognitive testing) compared with no cognitive impairment was associated with higher total Medicare expenditures after accounting for demographics and comorbidities.^19^ However, analyses did not examine the extent to which excess costs may be explained by functional impairments and frailty. Functional impairments and frailty both predict higher healthcare costs in community-dwelling older adults unselected for cognitive impairment^20–23^ and are associated with cognitive impairment among older adults.^24–26^ However, the degree to which they account for higher healthcare costs among individuals with cognitive impairment, with or without dementia, and after adjusting for demographics and comorbidities, is unknown. Addressing this question is the aim of the current study.

## METHODS

### Study Design

We studied community-dwelling older adults enrolled in four prospective cohort studies linked with each other and Medicare claims who completed a comprehensive index examination. Respectively, these index examinations occurred at the year 16 (2002 to 2004) visit for the Study of Osteoporotic Fractures (SOF), year 7 (2007 to 2009) visit for the Osteoporotic Fractures in Men (MrOS) study, year 6 (2002 to 2003) visit for the Health, Aging and Body Composition Study (HABC), and 2011 visit for the National Health and Aging Trends Study (NHATS) (Table 1).

### Participants

Of the 17,282 women and men from the four cohorts who completed an index examination, 1236 (7.2%) were excluded for missing data on demographics, lifestyle variables, cognitive testing, self-reported functional status, or phenotypic frailty, and 7881 (45.6%) were excluded for not being continuously enrolled in fee-for-service Medicare from 12 months before until 36 months after their index examination or until death within this period. The remaining 8165 individuals (4318 women and 3847 men) formed the analytic cohort (Supplement Fig. 1).

### Independent Variable

Cognitive impairment was defined for MrOS, SOF, and HABC as either a self or proxy report of a clinician diagnosis of dementia or a cognitive test score (Mini-Mental State Examination for SOF, Modified Mini Mental State for MrOS and HABC) at least 1.5 standard deviations (SD) below the education level (< 9 years, 9–12 years, and > 12 years) stratified mean of adults aged 65–69 years. We chose the youngest age category as the reference group to limit its inclusion of persons with dementia. For NHATS, we used the NHATS algorithm to define cognitive impairment based on a self-or-proxy-reported clinician diagnosis of dementia combined with a score of > 2 on the proxy-completed 8-item Informant Interview to Differentiate Aging and Dementia (AD8) or brief cognitive test scores more than 1.5 SD below the mean in at least two of three cognitive domains: memory (immediate and delayed word recall), orientation, and executive function (clock drawing).

### Covariables

Comorbidity was defined by count of a subset of 25 non-dementia Chronic Condition Warehouse (CCW) medical conditions selected by the Centers for Medicare and Medicaid Services (CMS) to facilitate the study of chronic medical conditions in the Medicare population. We focused on conditions commonly associated with higher healthcare costs: anemia, chronic kidney disease, chronic obstructive pulmonary disease, depression, diabetes, heart failure, ischemic heart disease, osteoporosis, breast cancer (women only), colorectal cancer, lung cancer, endometrial cancer (women only), and prostate cancer (men only). We excluded diagnoses that are commonly asymptomatic or are risk factors for symptomatic disease (e.g., excluding hyperlipidemia, a risk factor for cardiovascular disease). We used Medicare claims data to identify the presence of each condition using diagnosis codes from existing CCW algorithms.

For secondary analyses, we used a weighted comorbidity measure (community version 12 [v1209.F1] of CMS-Hierarchical Condition Categories [CMS-HCC] model) for each participant that considers demographics (age, sex, Medicaid eligibility, and disability status) and over 14,000 diagnosis codes in inpatient and outpatient claims in the 12 months prior to the index examination. The CMS-HCC model is specifically calibrated to predict healthcare costs in Medicare beneficiaries.^27,28^ We did not use HCC as the primary comorbidity measure because its algorithm includes dementia diagnoses.

### Potential Explanatory Variables

Measures of functional impairments and frailty were harmonized across the four cohort studies.^23^ At their index examination, participants for each study were asked about their difficulty performing four activities of daily living (walking a few blocks on level ground, climbing up 10–20 steps, transferring from bed to chair, and bathing or showering). Participants were categorized as having none (referent group), one, two, three, or four functional impairments.

Phenotypic frailty was assessed for each participant at their index examination using a harmonized version^23^ of five components initially proposed by Fried and colleagues,^29^ including shrinking (recent weight loss of ≥ 5% or ≥ 10 pounds or BMI < 18.5 kg/meters-squared),^30^ weakness (grip strength < 32 kg for men or < 20 kg for women),^31^ self-reported poor energy, slowness (gait speed < 0.8 m/s for men or < 0.6 m/s for women or use of a walking aid),^30,32,33^ and low physical activity (reporting never walking for exercise and not engaging in moderate or vigorous activity).^34^ Participants were categorized as robust (no components, referent group), having phenotypic pre-frailty (1 or 2 components), or having phenotypic frailty (≥ 3 components). The Kim claims-based frailty index (CFI)^35^ approximating the deficit accumulation index of frailty^36,37^ was calculated using diagnosis and procedure codes in Medicare claims files in the year prior to the index examination. The CFI is a continuous measure with a potential range of 0 to 1.

### Dependent Variable

The outcome variable, standardized annualized total direct healthcare costs for 36 months following the index examination, was calculated from the healthcare sector perspective^38,39^ and included costs paid by Medicare or supplementary insurance and patient out-of-pocket payments. Costs were calculated as the sum of allowable charges for acute hospital stays, inpatient rehabilitation facility stays, skilled nursing facility (SNF) stays paid under Medicare part A, outpatient care, durable medical equipment, and home healthcare (HHC). We selected a 3-year follow-up for healthcare costs because the substantial year-to-year variability in healthcare costs and the noisiness of cost data are dampened with longer follow-up.^40^ For those who died before the end of the 3-year follow-up period, annualized costs were calculated as cumulative costs divided by the duration of the truncated follow-up period. Standardized costs were calculated using previously published and validated methods.^41–43^ Costs of all units of utilization were adjusted for healthcare cost inflation to US 2023 dollars.^43^

### Statistical Analysis

As specified a priori, we stratified all analyses by sex because, compared with men, women have higher dementia prevalence,^44,45^ greater frailty prevalence,^46,47^ report more functional impairments,^48,49^ and have higher US per capita healthcare spending.^50^ Thus, we theorized that associations of dementia, frailty, functional impairment, and Medicare expenditures may vary by sex. Analyses stratified by sex are agnostic as to whether model parameters are similar in men and women.

Baseline characteristics were reported descriptively for participants with and without cognitive impairment.

Generalized linear models with log link and gamma variance were used to estimate the multivariable-adjusted (age, race, geographic region, comorbidities) associations of cognitive impairment with mean annualized total healthcare costs. Models were not adjusted for education because educational level was part of the definition of cognitive impairment. From these models, we used margins to predict mean annualized total healthcare costs by cognitive impairment status.

We performed analyses for marginal incremental costs to distinguish without and with adjustment for, respectively, functional impairments, phenotypic frailty, a claims-based deficit accumulation frailty index, or a combination of all three. In each case, we decomposed the total effect of cognitive impairment on mean annualized incremental total healthcare costs into two separate components: direct and indirect. For example, for functional impairments, we estimated total incremental costs of cognitive impairment from a base model adjusted for age, race, geographic region, and comorbidity count. We then estimated direct incremental costs of cognitive impairment from a model further adjusted for functional impairments (Functional Impairments-Adjusted Model). Finally, we estimated indirect incremental costs of dementia attributable to functional impairments by subtracting incremental costs of dementia for the Functional Impairments-Adjusted Model from those of the Base Model. We followed a similar approach to separately estimate direct and indirect incremental costs of cognitive impairment attributable to phenotypic frailty, the claims-based deficit accumulation frailty index, and to the combination of functional impairments, phenotypic frailty, and the claims-based deficit accumulation frailty index. We used bootstrap methods to find 95% confidence intervals for all estimates of indirect costs.

We performed two sensitivity analyses. First, we substituted an alternative comorbidity measure, whereas our primary comorbidity measure, the count of CCW conditions, does not include dementia codes, and our alternative comorbidity measure, the weighted CMS-HCC multimorbidity measure, includes dementia codes. Second, we performed analyses pooling the male and female group estimates, first with equal group weights and, alternatively, weighting by group sample size.

## RESULTS

The analysis cohort comprised 8165 community-dwelling, older women (52.9%) and men (47.1%) (Table 2). The prevalence of cognitive impairment was 12.1% in women and 10.9% in men. Compared to women and men without cognitive impairment, those with cognitive impairment were older, less likely to be White, had more medical conditions, a higher prevalence of phenotypic frailty and functional impairments, and more often were hospitalized, admitted for post-acute care, and died during study follow-up.

Incremental THC for cognitive impairment after adjustment for age, race, geographic region, and comorbidities were $6883 (95% CI, 3461 to 10,305) in women and $7276 (3298 to 11,254) in men (Table 3). These results were derived from the difference between adjusted mean THC with cognitive impairment ($21,005 [95% CI, 17,690 to 24321]) versus without cognitive impairment ($14,122 [13,197 to 15,048]) in women, and between the adjusted mean THC with cognitive impairment ($22,127 [18,269 to 25,985]) versus without cognitive impairment ($14,851 [13,769 to 15,933]) in men (Supplemental Table 1).

**Figure 1:**
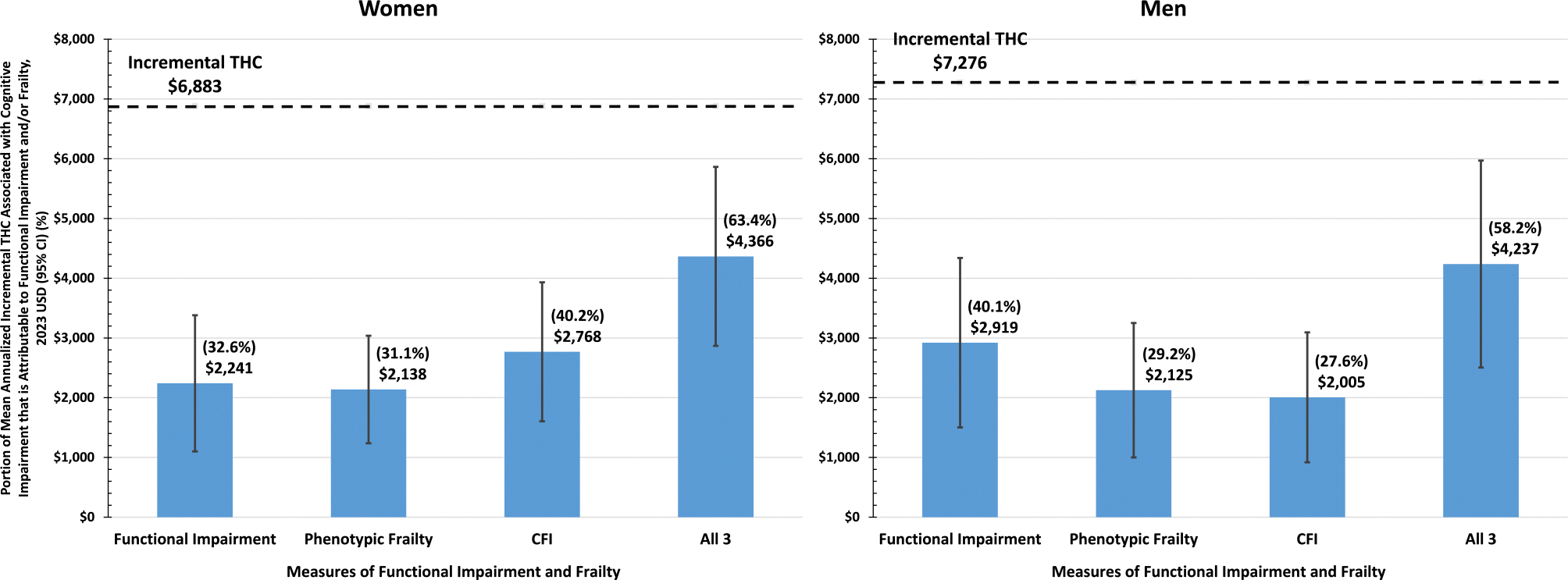
Mean annualized incremental total health care costs associated with cognitive impairment* that are explained by functional impairments and frailty, 2023 USD (95% CI). CFI, claims-based deficit accumulation frailty index; CI, confidence intervals; THC, total health care costs; USD, United States dollars. *Total incremental costs attributable to cognitive impairment (i.e., $6882 in women and $7276 in men) are adjusted for age, race, geographic region, and chronic medical conditions.

In women, of the $6883 greater annualized incremental THC in those with versus without cognitive impairment, $2241 (32.6%) was estimated as indirect and attributable to functional impairments, $2138 (31.1%) as attributable to phenotypic frailty, $2768 (40.2%) as attributable to CFI, and $4366 (63.4%) was accounted for when all three candidate explanatory variables were considered in combination (Table 3, Fig. 1). Similarly, in men, of the $7276 greater annualized incremental THC in those with versus without cognitive impairment, $2919 (40.1%) was estimated as indirect and attributable to functional impairments, $2125 (29.2%) as attributable to phenotypic frailty, $2005 (27.6%) as attributable to CFI, and $4237 (58.2%) was accounted for when all three candidate explanatory variables were considered in combination.

In sensitivity analyses substituting the CMS-HCC multimorbidity measure as the measure of comorbidity burden, incremental THC for cognitive impairment was $5051 (1907 to 8194) in women and $6065 (2330 to 9800) in men (Supplemental Table 2). In women, of the $5051 greater annualized incremental THC in those with versus without cognitive impairment, $1320 (26.1%) was estimated as indirect and attributable to functional impairments, $1439 (28.5%) as attributable to phenotypic frailty, $1344 (26.6%) as attributable to CFI, and $2588 (51.2%) was accounted for when all three candidate explanatory variables were considered in combination. Similarly, in men, of the $6065 greater annualized incremental THC in those with versus without cognitive impairment, $2099 (34.6%) was estimated as indirect and attributable to functional impairments, $1285 (21.2%) as attributable to phenotypic frailty, $1102 (18.2%) as attributable to CFI, and $2805 (46.2%) was accounted for when all three candidate explanatory variables were considered in combination. In the sensitivity analyses pooling group results for men and women, the magnitude of excess healthcare costs associated with cognitive impairment was intermediate between the separate results for men and women, and the confidence intervals for the combined group were narrower (Supplemental Table 3).

## DISCUSSION

In a large cohort of community-dwelling older Medicare beneficiaries, we found that, after accounting for demographic variables and comorbidities, about 60% of the incremental total direct healthcare costs associated with cognitive impairment were attributable to a higher prevalence of functional impairments and frailty among individuals with cognitive impairment.

Results of the current study were consistent with prior studies that found that functional impairments are associated with increased costs in individuals with dementia. First, in several cross-sectional analyses of individuals with dementia, none of which included a non-dementia control group, greater impairment in instrumental activities of daily living (IADLs) and ADLs was associated with increased Medicare expenditures,^17^ increased societal costs,^51,52^ and increased direct, indirect, and total costs,^53,54^ respectively. Similarly, a small prospective study of individuals with dementia, which also did not include a non-dementia control group, found that a higher dependence score was independently associated with higher subsequent total healthcare costs.^55^ Finally, the prospective Epidemiological Study of the Elderly (H-EPESE), which examined healthcare utilization in Mexican-American Medicare beneficiaries with (*n* = 193) and without (*n* = 855) dementia, reported that increased odds of emergency room admissions and hospitalizations associated with dementia were significantly attenuated after controlling for comorbidities and ADL limitations.^14^ The current study adds to these earlier studies by confirming the impact of functional impairments on cognitive impairment-associated healthcare costs in a larger, controlled, prospective, and more geographically and ethnically diverse multi-cohort population of older men and women.

By contrast, we found no prior studies that specifically analyzed, as we did, whether or the extent to which frailty accounts for increased cognitive impairment-associated healthcare costs. Also of importance, our study included measures of frailty defined using the two most widely used frailty frameworks: the frailty phenotype, in which frailty is manifested by markers of limited physiologic reserve across several organ systems, and the deficit accumulation model that views frailty as the accumulation of multiple health deficits.

Current study results may have implications for clinical practice. If functional impairments and frailty account for most excess healthcare costs associated with cognitive impairment, it is possible that interventions that focus on modifiable aspects of functional impairments and frailty will mitigate these excess healthcare costs. These may include referral to a nutritionist, exercise programs, or physical or occupational therapy;^56^ comprehensive geriatric assessment;^57^ or multidisciplinary community or home-based interventions^58,59^ intended to prevent or delay progression to severe frailty and overt disability. At minimum, current results strongly suggest a line for future research, investigation into whether interventions to improve or slow the decline in function and worsening in frailty in patients with cognitive impairment will lower healthcare costs by a greater amount than the costs of the intervention, while also considering the patient burden and acceptance of cognitive assessment.

Our study has numerous strengths. These include its large and geographically and ethnically diverse analysis cohort; prospective design; cognitive phenotyping of participants using cohort study data; harmonization of cognitive impairment, frailty, and functional impairments measures across cohorts; cross-cohort linkage of participants to their Medicare claims; and confirmation of main study findings in sensitivity analyses. However, there are several limitations. These include that pharmacy costs, costs of long-term SNF stays not paid under Medicare part A, and indirect costs of care not captured in the Medicare claims (such as informal care from family and friends) are not included. Second, the analysis cohort was community-dwelling, and 79% of participants were White and non-Hispanic, so results may not be generalizable to other race and ethnicity groups. Third, it is unclear whether the extent to which frailty and functional impairments accounted for excess healthcare costs in study participants with cognitive impairment differs depending on whether individuals had self-or-proxy reported dementia alone, impaired cognitive testing alone, both, or varies depending on the severity of cognitive impairment. Fourth, because functional impairment and frailty were measured at the same time as cognitive function, our analyses could not distinguish the order in which these conditions developed in participants. Fifth, because MrOS, SOF, and NHATS did not collect updated information on cognitive impairment, functional impairments, and frailty status during the three years after their study index examinations, we could not evaluate whether changes in these measures during this interval affected results. Sixth, because functional impairment, phenotypic frailty, and CFI partially overlap, we could not completely disentangle the extent to which they independently account for excess cognitive impairment-associated healthcare costs. Seventh, annualization of healthcare costs in individuals whose follow-up was shortened by death, which may have been more likely in individuals with cognitive impairment, may have overestimated total healthcare costs in this group. However, it is unknown whether this affected the extent to which functional impairment and frailty independently account for excess cognitive impairment-associated healthcare costs. Finally, these are observational data and do not establish that interventions that improve or reduce the progression of frailty and functional impairments will lower healthcare costs in this population.

In conclusion, in both male and female Medicare beneficiaries, after accounting for demographics and comorbidities, most of the incremental THC associated with cognitive impairment was attributable to functional impairments and frailty. These results were similar for women and men. Future studies should investigate whether addressing these geriatric syndromes will mitigate their impact on THC among community-dwelling older adults with cognitive impairment.

## Supplementary Material

supplement

**Supplementary Information** The online version contains supplementary material available at https://doi.org/10.1007/s11606-025-10073-z.

## Figures and Tables

**Table 1:** Cohort Study Information

Cohort*	Demographics	Geographic region	Eligibility criteria	Baseline exam	Index exam

SOF	9704 community-dwelling white women, and 662 community-dwelling black women	4 US sites (Baltimore, MD; Minneapolis, MN; Pittsburgh, PA; Portland, OR)	Women aged 65 and older, ability to walk without the assistance of another person, and absence of bilateral hip replacement	1986–1988 white cohort; 1997–1998 black cohort	Year 16 (2002–2004) assessment of cognitive function, frailty phenotype, and self-reported functional impairments
MrOS	5994 community-dwelling men	6 US sites (Birmingham, AL; Minneapolis, MN; Palo Alto, CA; Pittsburgh, PA; Portland, OR; San Diego, CA)	Men aged 65 and older, ability to walk without the assistance of another person, and absence of bilateral hip replacement	2000–2002	Year 7 (2007–2009) assessment of cognitive function, frailty phenotype, and self-reported functional impairments
HABC	3075 black and white community-dwelling men and women	2 US sites (Memphis, TN and Pittsburgh, PA)	Aged 70–79 with no self-report of mobility difficulty or mobility disability	1997–1998	Year 6 (2002–2003) assessment of cognitive function, frailty phenotype, and self-reported functional impairments
NHATS	8245 men and women (7609 community-dwelling)	US nationally representative, population-based sample	Medicare beneficiary aged 65 and older	2011; replenishment cohort 2015	2011 assessment of cognitive function, frailty phenotype, and self-reported functional impairments

*SOF* Study of Osteoporotic Fractures, *MrOS* Osteoporotic Fractures in Men Study, *HABC* Health, Aging and Body Composition Study, *NHATS* National Health and Aging Trends Study

* Institutional review boards at each participating site reviewed and approved the four cohort studies; all participants provided written informed consent

**Table 2:** Characteristics of Participants by Cognitive Impairment Status*

	Cognitive impairment	No cognitive impairment

**Women**	***n* = 521**	***N* = 3797**
Age, years, mean (SD)	83.6 (6.2)	79.5 (6.4)
Race, *n* (%)		
Non-Hispanic White	305 (58.5)	3005 (79.1)
Non-Hispanic Black	175 (33.6)	675 (19.2)
Other	41 (7.9)	117 (3.1)
Number of medical conditions†, mean (SD)	1.8 (1.5)	1.4 (1.4)
CMS-HCC score, mean (SD)	1.6 (1.1)	1.2 (0.9)
Functional impairments, *n* (%)		
None	133 (25.1)	2065 (54.4)
1–2	163 (31.3)	1278 (33.7)
3–4	225 (43.2)	454 (12.0)
Phenotypic frailty, *n* (%)	313 (60.1)	1167 (30.7)
CFI, mean (SD)	0.21 (0.07)	0.16 (0.05)
Annualized THC, USD, mean (SD)	$23,883 (38,045)	$13,735 (24,124)
Hospitalized during follow-up, *n* (%)	319 (61.2)	1737 (45.7)
PAC stay during follow-up, *n* (%)	138 (26.5)	591 (15.6)
Died during follow-up, *n* (%)	170 (32.6)	404 (10.6)
**Men**	***n* = 418**	***n* = 3429**
Age, years, mean (SD)	81.6 (6.0)	78.0 (5.9)
Race, *n* (%)		
Non-Hispanic White	256 (61.2)	2858 (83.3)
Non-Hispanic Black	127 (30.4)	403 (11.8)
Other	35 (8.4)	168 (4.9)
Number of medical conditions†, mean (SD)	1.8 (1.6)	1.3 (1.4)
CMS-HCC score, mean (SD)	1.7 (1.3)	1.2 (1.0)
Functional impairments, *n* (%)		
None	172 (41.1)	2448 (71.4)
1–2	120 (28.7)	748 (21.8)
3–4	126 (30.1)	233 (6.8)
Phenotypic frailty, *n* (%)	169 (40.4)	564 (16.4)
CFI, mean (SD)	0.18 (0.07)	0.15 (0.05)
Annualized THC, USD, mean (SD)	$28,393 (50,105)	$14,138 (27,201)
Hospitalized during follow-up, *n* (%)	253 (60.5)	1465 (42.7)
PAC stay during follow-up, *n* (%)	98 (23.4)	305 (8.9)
Died during follow-up, *n* (%)	132 (31.6)	374 (10.9)

*CCW* Chronic Condition Warehouse, *CFI* Kim Claims-based Frailty Index, *CMS-HCC* Centers for Medicare & Medicaid Services-Hierarchical Condition Categories, *IQR* interquartile range, *PAC* post-acute care, *SD* standard deviation, *THC* total healthcare costs, *USD* 2023 US dollars

* All participant characteristics presented above were significantly different between individuals with versus without cognitive impairment at *p* < 0.001

† Number of CCW conditions: anemia, chronic kidney disease, chronic obstructive pulmonary disease, depression, diabetes, heart failure, ischemic heart disease, osteoporosis, breast cancer (women only), colorectal cancer, lung cancer, prostate cancer (men only), and endometrial cancer (women only)

**Table 3:** Mean Annualized Incremental Total Health Care Costs Associated with Cognitive Impairment that are Explained by Functional Impairments and Frailty, 2023 USD (95% CI)

Model name	Model description	Women	Men

Base*	Adjusted for age, race, geographic region, and comorbidity count	6883 (3461, 10,305)	7276 (3298, 11,254)
Base + functional impairments†	Independent of functional impairments (direct)	4642 (1433, 7851)	4356 (660, 8053)
	Related to functional impairments (indirect)	2241 (1101, 3381)	2919 (1501, 4338)
Base + phenotypic frailty‡	Independent of phenotypic frailty (direct)	4745 (1667, 7823)	5151 (1373, 8929)
	Related to phenotypic frailty (indirect)	2138 (1238, 3038)	2125 (1000, 3251)
Base + CFI	Independent of CFI (direct)	4115 (867, 7363)	5271 (1465, 9076)
	Related to CFI (indirect)	2768 (1604, 3932)	2005 (918, 3093)
Base + functional impairments + phenotypic frailty + CFI	Independent of all 3 variables (direct)	2518 (−477, 5513)	3039 (−570, 6648)
	Related to functional impairments, frailty, and/or CFI (indirect)	4366 (2867, 5864)	4237 (2504, 5970)

*CCW* Chronic Condition Warehouse, *CFI* claims-based deficit accumulation frailty index, *CI* confidence intervals, *USD* 2023 U.S. dollars

* Base and all subsequent models were adjusted for age, race, geographic region, and comorbidities as a count of the number of CCW conditions (up to 12 in women or up to 11 in men). Referent group are those without cognitive impairment

† Functional impairments categorized as none, 1, 2, 3 or 4

‡ Phenotypic frailty categorized as robust, pre-frail, or frail

## Data Availability

SOF data are available to the public via SOF online (https://sofonline.ucsf.edu). MrOS data are available to the public via MrOS online (https://mrosonline.ucsf.edu). HABC data are available to the public via HABC online (https://healthabc.nia.nih.gov). NHATS data are available to the public via NHATS online (www.nhats.org). Medicare claims data for participants in the SOF, MrOS, HABC, and NHATS studies are not publicly available. CMS enters into data use agreements with data requesters for disclosures of protected health information and/or personally identifiable information to ensure that data requesters adhere to CMS privacy and security requirements and data release policies. These data are available for purchase from CMS after execution of an approved data use agreement. See https://resdac.org for more information.
