# Supplementary material for: Effects of Functional Impairments and Frailty on the Association of Cognitive Impairment with Total Healthcare Costs: A Prospective Multi-cohort Study
Source: J Gen Intern Med. Author manuscript; Available in PMC 2026 Apr 6. (PMC13051473; doi:10.1007/s11606-025-10073-z)
Supplement: supplement [file NIHMS2152664-supplement-supplement.docx]

**Appendix: Supplemental Figure and Tables**

**Supplemental Figure 1. Participant Flow Diagram**

**
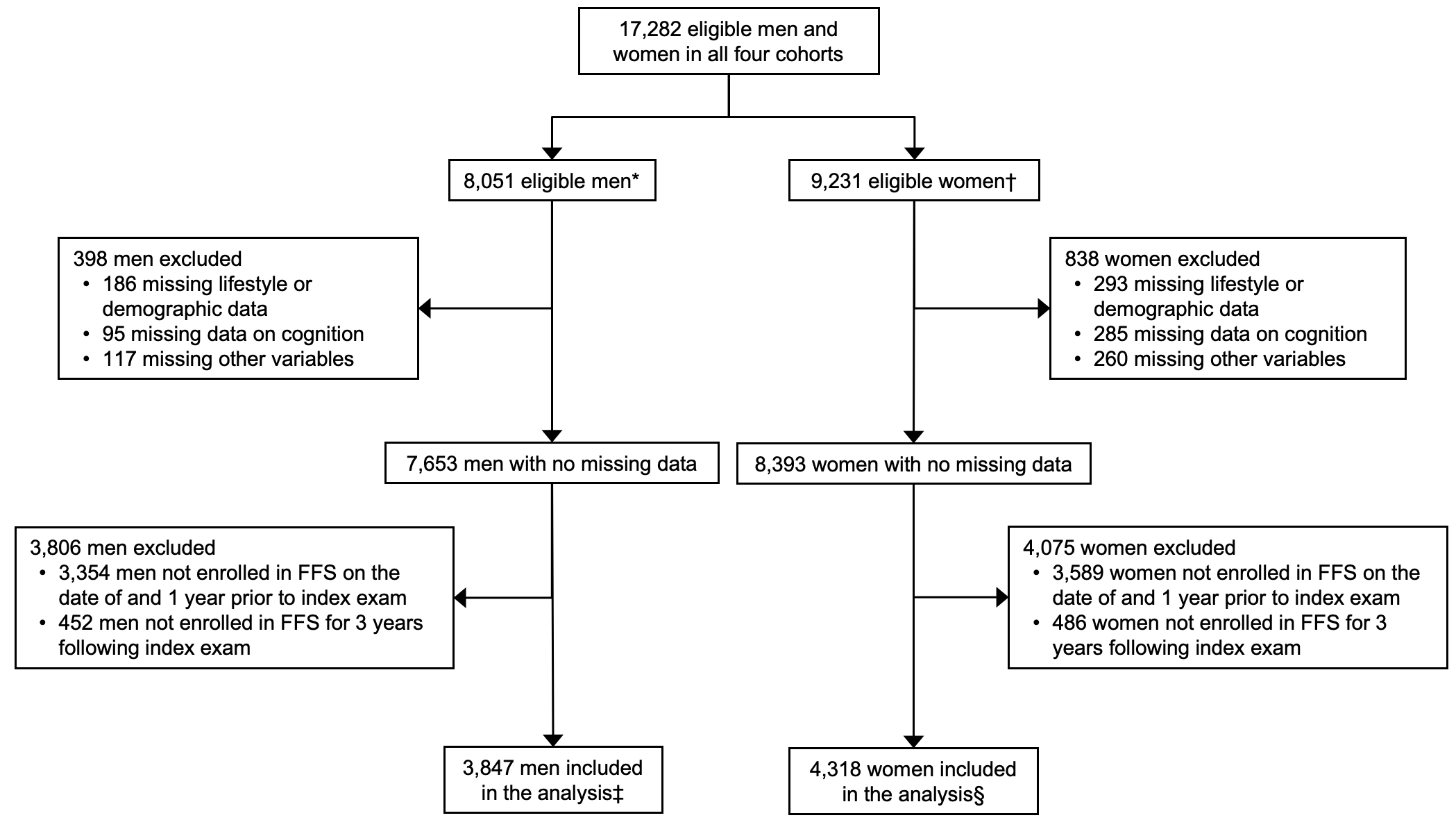
**

* Of these men, 3790 were enrolled in MrOS; 1211 were enrolled in Health ABC; and 3050 were enrolled in NHATS.

† Of these women, 3676 were enrolled in SOF; 1408 were enrolled in Health ABC; and 4147 were enrolled in NHATS.

‡ Of these men, 1556 were enrolled in MrOS; 638 were enrolled in Health ABC; and 1653 were enrolled in NHATS.

§ Of these women, 1371 were enrolled in SOF; 703 were enrolled in Health ABC; and 2244 were enrolled in NHATS**.**

**Supplemental Table 1. Predicted mean annualized total health care costs by cognitive status without and with adjustment for functional impairments and/or frailty, 2023 USD (95%CI)**

|  | **Women** | | **Men** | |
| --- | --- | --- | --- | --- |
|  | **No cognitive impairment** | **Cognitive impairment** | **No cognitive impairment** | **Cognitive impairment** |
| Base Model^*^ | 14122 (13197, 15048) | 21005 (17690, 24321) | 14851 (13769, 15933) | 22127 (18269, 25985) |
| Base Model + functional impairments† | 14271 (13340, 15203) | 19016 (16088, 21944) | 15110 (13956, 16263) | 20261 (16665, 23856) |
| Base model + phenotypic frailty‡ | 14322 (13340, 15305) | 18965 (15924, 22005) | 15314 (14106, 16521) | 19670 (16183, 23157) |
| Base model + CFI | 14536 (13482, 15591) | 18652 (15581, 21722) | 15131 (13968, 16294) | 20402 (16768, 24036) |
| Base model + functional impairments + phenotypic frailty + CFI | 14650 (13596, 15703) | 17167 (14405, 19930) | 15538 (14259, 16816) | 18577 (15226, 21927) |

CCW = Chronic Condition Warehouse, CFI = claims-based deficit accumulation frailty index, CI = confidence intervals, USD = 2023 U.S. dollars

^*^ Adjusted for age, race, geographic region, and multimorbidity as a count of the number of CCW conditions (up to 12 in women or up to 11 in men).

† Functional impairments categorized as none, 1, 2, 3 or 4.

‡ Frailty phenotype categorized as robust, pre-frail or frail.

**Supplemental Table 2. Sensitivity Analysis^1^ of Mean Annualized Incremental Total Health Care Costs Associated with Cognitive Impairment that are Explained by Functional Impairments and Frailty, Adjusted for HCC Score instead of CCW Count, 2023 USD (95% CI)**

| **Model Name** | **Model Description** | **Women** | **Men** |
| --- | --- | --- | --- |
| Base^*^ | Adjusted for age, race, geographic region, and HCC measure | 5051 (1907, 8194) | 6065 (2330, 9800) |
| Base + functional impairments† | Independent of functional impairments (direct) | 3721 (666, 6796) | 3965 (430, 7500) |
|  | Related to functional impairments (indirect) | 1320 (500, 2139) | 2099 (903, 3295) |
| Base + phenotypic frailty‡ | Independent of phenotypic frailty (direct) | 3612 (700, 6524) | 4780 (1252, 8409) |
|  | Related to phenotypic frailty (indirect) | 1439 (760, 2117) | 1285 (439, 2135) |
| Base + CFI | Independent of CFI (direct) | 3706 (607, 6805) | 4962 (1298, 8626) |
|  | Related to CFI (indirect) | 1344 (612, 2077) | 1102 (355, 1850) |
| Base + functional impairments + phenotypic frailty + CFI | Independent of all 3 variables (direct) | 2462 (-473, 5398) | 3260 (-260, 6780) |
|  | Related to functional impairments, frailty, and/or CFI (indirect) | 2588 (1509, 3667) | 2805 (1400, 4210) |

CCW = Chronic Condition Warehouse, CFI = claims-based deficit accumulation frailty index, CI = confidence intervals, CMS-HCC = Centers for Medicare & Medicaid Services-Hierarchical Condition Categories, USD = 2023 U.S. dollars

^*^ In this sensitivity analysis, base and all subsequent models were adjusted for age, race, geographic region, and for comorbidity determined by the HCC score instead of comorbidity as determined by the count of CCW conditions. Referent group are those without cognitive impairment.

† Functional impairments categorized as none, 1, 2, 3 or 4.

‡ Phenotypic frailty categorized as robust, pre-frail or frail.

**Supplemental Table 3. Sensitivity Analyses of Mean Annualized Incremental Total Health Care Costs Associated with Cognitive Impairment that are Explained by Functional Impairments and Frailty, in Male and Female Groups Separate and Pooled, 2023 USD (95% CI)**

| **Model Name** | **Model Description** | **Women Alone** | **Men Alone** | **Men + Women, sample size weights** | **Men + Women, equal group weights** |
| --- | --- | --- | --- | --- | --- |
| Base* | Adjusted for age, race, geographic region, and CCW count | 6883 (3461, 10305) | 7276 (3298, 11254) | 7068 (4456, 9681) | 7080 (4467, 9692) |
| Base + functional impairments† | Independent of functional impairments (direct) | 4642 (1433, 7851) | 4356 (660, 8053) | 4507 (2070, 6945) | 4499 (2062, 6936) |
|  | Related to functional impairments (indirect) | 2241 (1101, 3381) | 2919 (1501, 4338) | 2560 (1656, 3465) | 2580 (1676, 3484) |
| Base + phenotypic frailty‡ | Independent of phenotypic frailty (direct) | 4745 (1667, 7823) | 5151 (1373, 8929) | 4936 (2514, 7359) | 4948 (2526, 7370) |
|  | Related to phenotypic frailty (indirect) | 2138 (1238, 3038) | 2125 (1000, 3251) | 2132 (1416, 2848) | 2132 (1416, 2847) |
| Base + CFI | Independent of CFI (direct) | 4115 (867, 7363) | 5271 (1465, 9076) | 4660 (2169, 7150) | 4693 (2203, 7183) |
|  | Related to CFI (indirect) | 2768 (1604, 3932) | 2005 (918, 3093) | 2409 (1611, 3206) | 2387 (1589, 3184) |
| Base + functional impairments + phenotypic frailty + CFI | Independent of all 3 variables (direct) | 4366 (2867, 5864) | 4237 (2504, 5970) | 4305 (3164, 5446) | 4302 (3161, 5442) |
|  | Related to combined 3 variables (indirect) | 2518 (-477, 5513) | 3039 (-570, 6648) | 2763 (431, 5096) | 2779 (446, 5111) |

CCW = Chronic Condition Warehouse, CFI = claims-based deficit accumulation frailty index, CI = confidence intervals, CMS-HCC = Centers for Medicare & Medicaid Services-Hierarchical Condition Categories, USD = 2023 U.S. dollars

* Base and all subsequent models were adjusted for age, race, geographic region, and for comorbidity determined by the count of CCW conditions. Referent group are those without cognitive impairment.

† Functional impairments categorized as none, 1, 2, 3 or 4.

‡ Phenotypic frailty categorized as robust, pre-frail or frail.
